# Difenoconazole-Loaded Nanostructured Lipid Carriers: Preparation, Characterization, and Evaluation

**DOI:** 10.3390/ph18060780

**Published:** 2025-05-23

**Authors:** Yinghong Li, Hu Zhang, Tingting Meng, Yuqin Zhou, Beilei Zhou, Shihan Du, Hong Yuan, Fuqiang Hu

**Affiliations:** 1 College of Pharmaceutical Science, Zhejiang University, No. 866 Yuhangtang Road, Hangzhou 310058, China; 2 Zhejiang Institute for Food and Drug Control, NMPA Key Laboratory for Testing and Warning of Pharmceutical Microbiology, Zhejiang Key Laboratory of Biopharmaceutical Contact Materials, No. 325 Pingle Street, Hangzhou 310052, China; 3 Institute of Agro-Product Safety and Nutrition, Zhejiang Academy of Agricultural Sciences, No. 198 Shiqiao Road, Hangzhou 310021, China; 4 School of Pharmacy, China Pharmaceutical University, No. 639, Longmian Avenue, Nanjing 211198, China

**Keywords:** nanostructured lipid carriers, difenoconazole, preparation, antifungal effect, zebrafish, toxicity

## Abstract

**Background/Objectives**: Difenoconazole (DFC) is a broad-spectrum fungicide. However, its application is limited due to poor aqueous solubility. Drugs with low solubility can be better absorbed using nanostructured lipid carriers (NLCs). Hence, the application of DFC in an NLC delivery system is proposed. **Methods**: Difenoconazole-loaded nanostructured lipid carriers (DFC-NLCs) with different solid–liquid lipid ratios were prepared by solvent diffusion method. Key physicochemical parameters, including particle diameter, surface charge (zeta potential), drug encapsulation efficiency, and morphological characteristics, were systematically characterized. Using Rhizoctonia solani (*R. solani*) as the model strain, inhibitory efficiency of DFC-NLC dispersion was compared with that of commercial dosage forms, such as 25% DFC emulsifiable concentrate (DFC-EC) and 40% DFC suspension concentrate (DFC-SC). Additionally, uptakes of DFC-NLC dispersions in *R. solani* were further observed by fluorescence probe technology. The safety profiles of DFC-NLCs and commercial dosage forms were evaluated using zebrafish as the model organism. Acute toxicity studies were conducted to determine the maximum non-lethal concentration (MNLC) and 10% lethal concentration (LC_10_). Developmental toxicity studies were performed to observe toxic phenotypes. **Results**: DFC-NLC dispersions were in the nanometer range (≈200 nm) with high zeta potential, spherical in shape with encapsulation efficiency 69.1 ± 1.8%~95.0 ± 2.6%, and drug loading 7.1 ± 0.3%~9.7 ± 0.6% determined by high-performance liquid chromatography coupled with tandem mass spectrometry (HPLC-MS/MS). Compared with commercial dosage forms, the antifungal effect of the DFC-NLC on *R. solani* was significantly improved in in vitro antibacterial experiments (*p* < 0.05). The 50% effective concentration (EC_50_) values were 0.107 mg·L^−1^ (DFC-NLC), 0.211 mg·L^−1^ (DFC-EC), and 0.321 mg·L^−1^ (DFC-SC), respectively. The uptakes of FITC-labeled DFC-NLC demonstrated that an NLC was appropriate to deliver DFC into pathogen to enhance the target effect. In safety assessment studies, DFC-NLCs exhibited a superior safety profile compared with commercial formulations (*p* < 0.05). **Conclusions**: This study investigates the feasibility of NLCs as delivery systems for poorly water-soluble fungicides, demonstrating their ability to enhance antifungal efficacy and reduce environmental risks.

## 1. Introduction

Pesticides are indispensable for mitigating biotic threats to crops, including phytopathogens, pests, and invasive weeds. However, more than 90% of the traditional pesticides have some defects, such as poor dispersion and dust drift [1]. In practice, pesticides that can not be fully absorbed and utilized by plants might run off into the environment, polluting the soil, air, and water sources and finally causing considerable damage to animals, plants, and human beings [2]. Recent advancements in nanotechnology have revolutionized agricultural practices [3,4]. With the aid of nanotechnology, nanopesticide delivery systems are constructed to provide new strategies for formulation processing and efficient utilization of poorly water-soluble pesticides [5,6,7,8,9]. There are several nano-formulations for classic nanopesticide delivery systems, such as nanoemulsions [10,11], nanocarriers for controlled release [12,13,14,15], inorganic metal and metal oxide nanoparticles with controlled sizes and shapes [16,17,18,19,20], and others. However, Kah et al. reported that there was about 20–30% median gain regarding the efficacy of nanoagrochemicals compared to conventional products based on previous research [21]. Thus, it could be seen that the efficacy of nanopesticide delivery systems to target organisms (such as pathogenic bacteria, insects, mites, weeds, rats, infected plants, etc.) failed to meet expectations of researchers.

Fast delivery as well as distribution of pesticide molecules in nanopesticide delivery systems to target organisms are important prerequisites to achieve antimicrobial effect. Ye et al. indicated the importance of root exudates in assessing the uptakes of gold nanoparticles coated with different charged ligands into rice plants [22]. Ye et al. reported that nano-hydroxyapatite was helpful for reducing the lead absorption of crops in Pb-contaminated soils [23]. Chinnaperumal et al. reported TiO_2_ nanoparticles exhibited significant antifeedant, larvicidal, and pupicidal activities on cotton bollworms, along with changed detoxifying glucosidase, carboxylesterase, and glutathione S-transferase levels in TiO_2_ nanoparticle-treated larvae of cotton bollworms [24]. These studies have provided evaluation methods for the transport and distribution of nanopesticides in plants [25]. The fact is that plant pathogens often interact with plants. Recognition and binding of the pathogen receptors on plant membranes at the cellular level result in parasitizing and infecting the host plants. The common concern points of diffusion, absorption, and transport of nanopesticide delivery systems in plants should focus on plant pathogens. Over the previous years, research on the mechanism of antifungal effects of nanopesticide delivery systems on pathogens mainly focuses on the effects of small size and large specific surface area [12], interfacial affinity effect [26,27], slow and controlled release effect [28,29], and high-efficiency transport effect [30]. However, the underlying molecular pathways were poorly characterized. Yet, further study remains to be conducted to decipher the inherent mechanism. In 2018, Zhang et al. reported a novel approach to probe the effects of fungicides on living fungal cells using fluorescent sensors [31], which allows us to visualize the antifungal effect of nanopesticide delivery systems on pathogens with bioinformatics methods.

Rice sheath blight is a worldwide rice disease, which is caused by infection of soil-borne fungus, *R. solani*. Rice sheath blight usually leads to a high level of yield loss both qualitatively and quantitatively [32,33]. Difenoconazole (DFC) is a triazole-type fungicide with annual sales exceeding 100 million USD. It is a sterol demethylation inhibitor, which can effectively prevent rice sheath blight. At 20 °C, its solubility in water is merely 3.3 mg·L^−1^. In the past few years, research on the formulation designs of DFC was mainly focused on suspension agent and water emulsion, especially on the selection and optimization of emulsifiers and thickeners [34,35]. Cao et al. reported that the droplet behavior of DFC-loaded mesoporous silica nanoparticles on the target leaf surface was a rather complex phenomenon [36]. However, DFC-loaded lipid nanoparticles with good bio-adhesion, permeability, and high specificity have not been reported.

An ideal nano-delivery system can enhance the distribution of drug molecules in target organisms, enhance bioavailability, and reduce toxicity [37,38,39]. Compared with a traditional nanoparticle delivery system, nanostructured lipid carriers (NLCs) have obvious advantages such as carriers with low cost, simple preparation processes, easy to be absorbed by the target organism, and low environmental risks [40,41,42,43,44,45,46]. Previous mechanistic investigations revealed that NLCs traverse epithelial barriers via energy-dependent endocytosis, exhibiting significantly higher cellular uptake than excretion rates [47]. When solid lipids are mixed with liquid lipids, the space capacity and stability of the delivery system are increased along with the accelerated drug delivery efficiency [48]. The working hypothesis tested for this study, based on the above research background, is that NLCs would offer a novel and alternative nanopesticide delivery system to improve the efficient utilization and reduce environmental risks.

This study employed a solvent diffusion approach to prepare difenoconazole-loaded nanostructured lipid carriers (DFC-NLCs). *R. solani* was used as the model strain, and inhibitory effectiveness of DFC-NLC dispersions were evaluated by laboratory inhibitory efficiency assessment. Delivery rules of DFC-NLC dispersions in plant pathogens were studied by fluorescence probe technology. Using zebrafish as the model organism, the safety profile of DFC-NLCs was evaluated through acute toxicity tests and developmental toxicity assays. This work aims to explore the application of DFC in an NLC nano-delivery system. Overall, this study provides new ideas, strategies, and approaches for formulation processing and efficient utilization of poorly water-soluble pesticides, which enrich and develop the theoretical and technical systems of nanotechnology in agriculture.

## 2. Results and Discussion

### 2.1. Selection of Lipid Materials

Monostearin, stearic acid, tristearin, and behenic acid glyceride were used as solid lipid materials; oleic acid was used as liquid lipid material; and DFC-NLC dispersions were prepared by solvent diffusion method. The obtained DFC-NLC dispersions appeared clear and transparent with light blue opalescence. However, along with storage time extended, the dispersions formed by solid–liquid lipid materials such as stearic acid–oleic acid, tristearin–oleic acid and behenic acid glyceride–oleic acid tended to coagulate or flocculate and had poor stability. For monostearin–oleic acid as solid–liquid lipid materials, the obtained DFC-NLC dispersions still kept stable at 5-day storage under 4 °C in the dark. However, short-term stability test was merely used to evaluate the stability performance of DFC-NLC dispersions during the experimental period. Long-term stability studies under ICH Q1A(R2) guidelines are critical for pharmaceutical formulation development. These studies should include rigorous evaluations under different controlled storage conditions, which will be performed in further studies.

In summary, comparative analysis of average diameter, polydispersity index, zeta potential, and short-term laboratory storage stability (Table 1) identified monostearin–oleic acid as the optimal lipid matrices, which were selected as lipid materials for subsequent investigations.

### 2.2. Characteristics of DFC-NLC Dispersions

**Figure 1:**
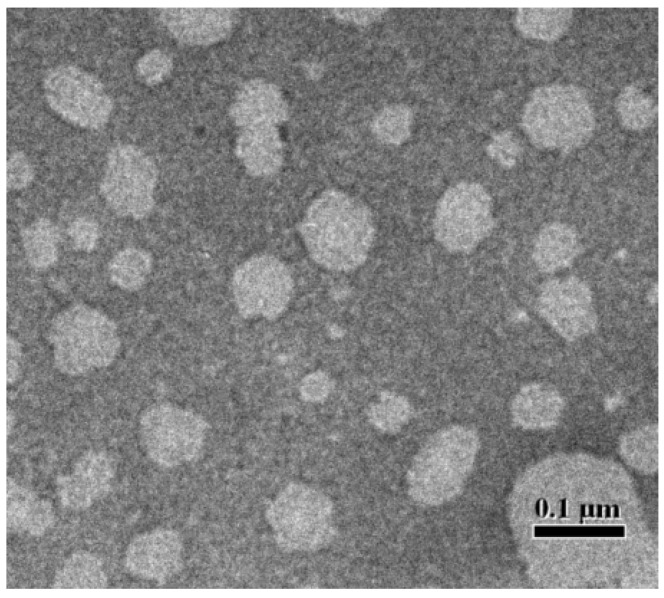
Shapes of DFC-NLCs prepared using monostearin–oleic acid as solid–liquid lipid materials with oleic acid proportion 30% under the observation of TEM.

Monostearin–oleic acid was used as solid–liquid lipid material, and the characteristics of the DFC-NLC dispersion formed from different solid–liquid lipid ratios of 10%, 20%, and 30% oleic acid are presented in Table 2. The results showed that DFC-NLC dispersions had high zeta potential in the nanometer range (about 200 nm), with entrapment efficiency values of 69.1 ± 1.8%~95.0 ± 2.6% and drug loading values of 7.1 ± 0.3%~9.7 ± 0.6% determined by HPLC-MS/MS. In addition, with the increase in oleic acid content, the particle size of DFC-NLCs decreased, while the absolute zeta potential, entrapment efficiency values, and drug loading values increased significantly. When solid and liquid lipid materials were mixed, criss-crossing space was formed. The higher the proportions of oleic acid were, the larger the formed effective drug loading space would be. In the follow-up studies, DFC-NLCs were prepared using monostearin as solid lipid material, oleic acid as liquid lipid material, and the oleic acid proportion was 30%. The DFC-NLC had spherical morphology under the observation of transmission electron microscopy (TEM) in Figure 1. The initial DFC concentration in DFC-NLC dispersion was 100 mg·L^−1^.

### 2.3. Antifungal Activity of DFC-NLC Dispersions

**Figure 2:**
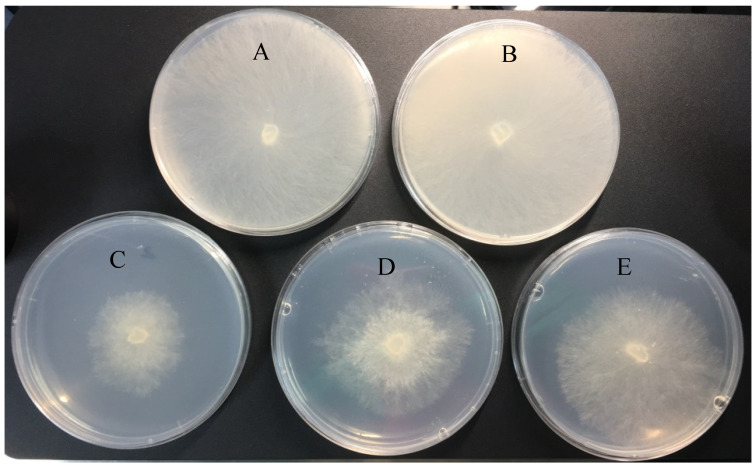
Images of the fungicidal activity of different formulations against *R. solani* after 48 h of culturing ((**A**) distilled water; (**B**) blank NLC dispersions; (**C**) DFC-NLC; (**D**) DFC-EC; and (**E**) DFC-SC).

Antifungal activities of three DFC formulations against *R. solani* were assessed through mycelial growth inhibition assays. The antifungal activities of DFC-NLC dispersions and the commercial dosage forms 25% DFC-EC and 40% DFC-SC were evaluated after 48 h of culturing (0.0156, 0.0313, 0.0625, 0.125, 0.250, 0.500, and 1 mg·L^−1^ active DFC concentration). The EC_50_ values for DFC-NLC dispersions, DFC-EC, and DFC-SC were 0.107, 0.211, and 0.321 mg·L^−1^, respectively. The fungicidal activity results shown in Table 3 indicate that the antifungal efficiency of DFC-NLC dispersions after 48 h of culturing is much stronger than that of the commercial dosage forms under the same conditions (*p* < 0.05). Structural design of NLCs may facilitate penetration through *R. solani* barriers [49,50]. Figure 2 visualizes the different inhibitory effects of DFC formulations on *R. solani* morphology after exposure of 48 h. Compared with DFC-EC and DFC-SC, DFC-NLC dispersions exhibited better fungicidal efficacy against *R. solani*. When treated with the blank NLC dispersion alone, the pathogen grew vigorously at 25 °C and covered the entire potato dextrose agar (PDA) plate, paralleling the growth patterns observed in aqueous controls, indicating that the blank NLC dispersion was safe and showed almost no obvious cytotoxicity.

### 2.4. Pathogen Uptakes of FITC-Labeled NLCs

**Figure 3:**
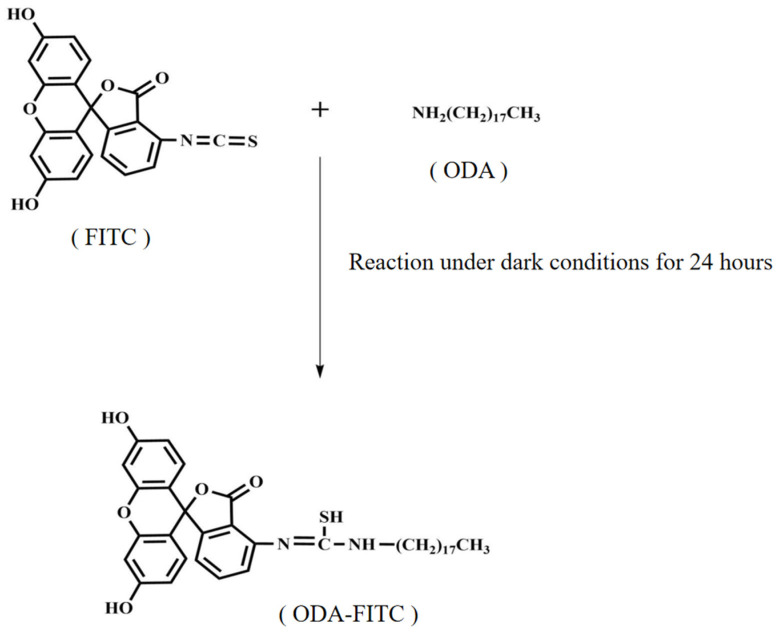
Covalent conjugation diagram of ODA-FITC synthesis.

Fluorescein isothiocyanate (FITC) can be coupled to other materials by binding to amino groups with the help of its reactive isothiocyanate group. In this experimental design, FITC was firstly bound to lipid material octadecylamine (ODA) to obtain octadecylamine fluorescein isothiocyanate graft (ODA-FITC) by covalent conjugation (Figure 3). The FITC-labeled NLC dispersion was then prepared and used as the fluorescent probe to characterize the uptake of nanoparticles in plant pathogens.

**Figure 4:**
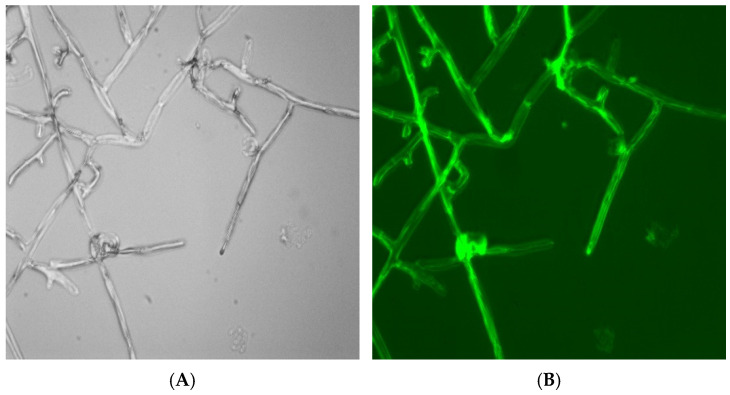
The growth situations after the pathogens were incubated with FITC-labeled NLCs without fluorescent excitation (**A**) and under fluorescent excitation (**B**).

The mycelium grew vigorously when it was observed under an inverted microscope without any fluorescence excitation (Figure 4A). Obvious fluorescence was observed inside the mycelium under fluorescent excitation (Figure 4B). The pesticide that loaded in the NLC might be released after being ingested by pathogens and then exert antifungal effects. The uptakes of FITC-labeled DFC-NLC experiments demonstrated that the NLC was appropriate to deliver DFC into pathogens to enhance the antifungal efficacy.

In the previous study, the research team demonstrated that nanostructured lipid carriers exhibit strong cell membrane uptake capacity [51]. In this section, DFC was wrapped in nano-formulation when the DFC-NLC was outside the fungal mycelia. Once DFC-NLC was ingested by pathogen, DFC might release from nano-formulation, thus exerting physiological activity.

### 2.5. Toxicity of DFC-NLC Dispersions

#### 2.5.1. Acute Toxicity

The effects of pesticide formulations on nontarget organisms are a critical determinant of environmental safety. Evaluation experiments were conducted using zebrafish as the research subject. As shown in Table 4, no zebrafish mortality is observed after 5 days exposure to DFC-NLC (DFC concentration 5.00 mg·L^−1^). However, at concentrations of 6.25, 7.50, 8.75, and 10.0 mg·L^−1^, the mortality percentages of zebrafish were 17%, 100%, 100%, and 100%, respectively. For the DFC-EC group, no mortality occurred at 1.25 and 2.50 mg·L^−1^, while exposure to 5.00, 10.0, and 20.0 mg·L^−1^ resulted in 33%, 100%, and 100% mortality percentages, respectively. Similarly, the DFC-SC group showed no mortality at 1.25 and 2.50 mg·L^−1^, but concentrations of 5.00, 10.0, and 20.0 mg·L^−1^ caused 60%, 100%, and 100% mortality percentages, respectively.

**Figure 5:**
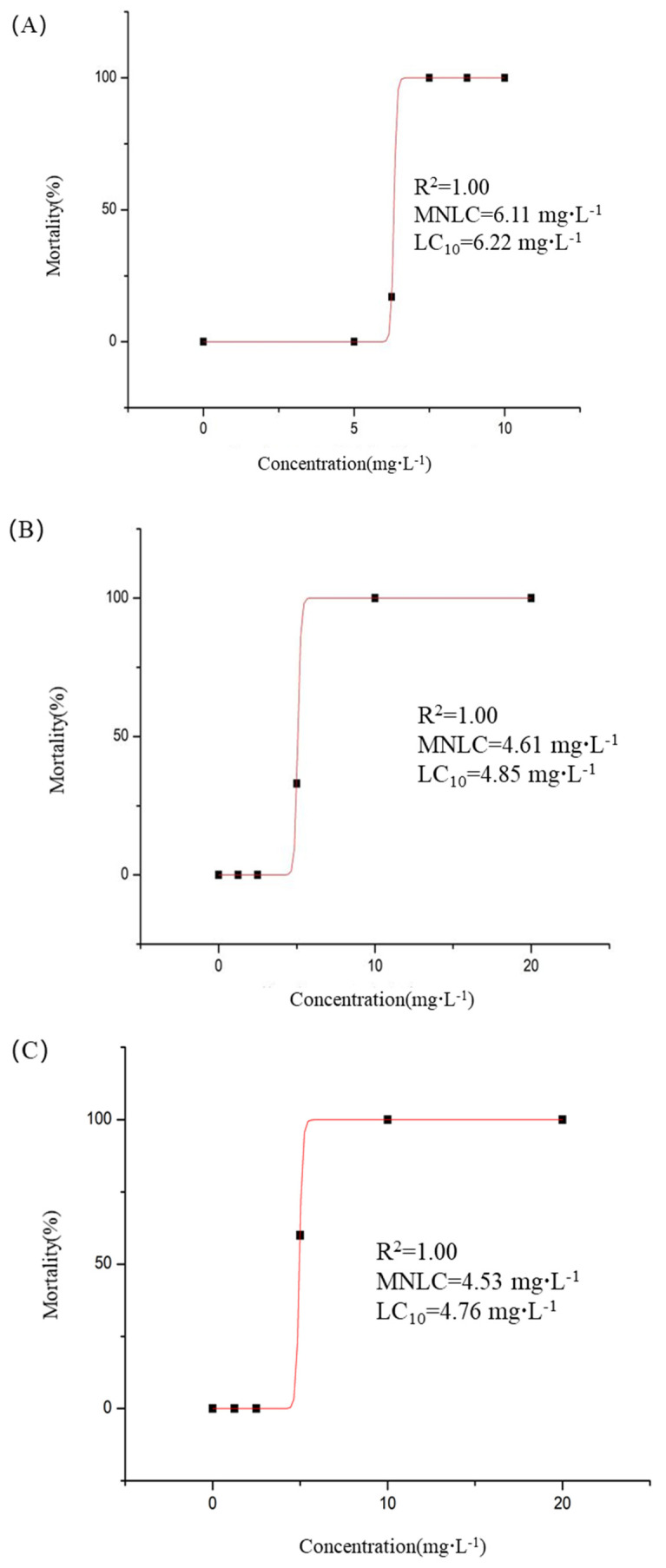
Mortality of zebrafish and Boltzmann fitting curves at 5 days ((**A**) DFC-NLC; (**B**) DFC-EC; and (**C**) DFC-SC).

Using Origin 8.0 software for statistical fitting, the maximum non-lethal concentration (MNLC) and 10% lethal concentration (LC_10_) of DFC-NLC for zebrafish were determined as 6.11 mg·L^−1^ and 6.22 mg·L^−1^, respectively (Figure 5A). For the emulsion formulation, the MNLC and LC_10_ of DFC-EC were 4.61 mg·L^−1^ and 4.85 mg·L^−1^ (Figure 5B), while the DFC-SC exhibited MNLC and LC_10_ values of 4.53 mg·L^−1^ and 4.76 mg·L^−1^ (Figure 5C), respectively. These results indicated that at equivalent concentrations of DFC, DFC-NLC induced significantly lower zebrafish mortality compared to both the emulsion and suspension formulations (*p* < 0.05).

#### 2.5.2. Developmental Toxicity

As illustrated in Table 5, DFC-NLC, DFC-EC, and DFC-SC all demonstrate developmental toxicity in zebrafish embryos compared to the blank control group. At the DFC concentration of 4.53 mg·L^−1^, both DFC-EC and DFC-SC induced multiple abnormalities in zebrafish embryos, including arrhythmia, slowed blood flow, shortened jaw, reduced eye size, liver shrinkage/absence, yolk sac absorption delay, intestinal deformity, trunk/tail/notochord curvature, decreased body length, behavioral abnormalities, reduced otic vesicles size, and absence of swim bladder. In contrast, the DFC-NLC group also caused arrhythmia, slowed blood flow, shortened mandible, reduced eye size, liver shrinkage/absence, delayed yolk sac absorption, shortened body length, behavioral abnormalities, reduced otic vesicles size, and absence of swim bladder. The DFC-NLC group did not induce intestinal abnormalities or trunk/tail/notochord curvature.

**Figure 6:**
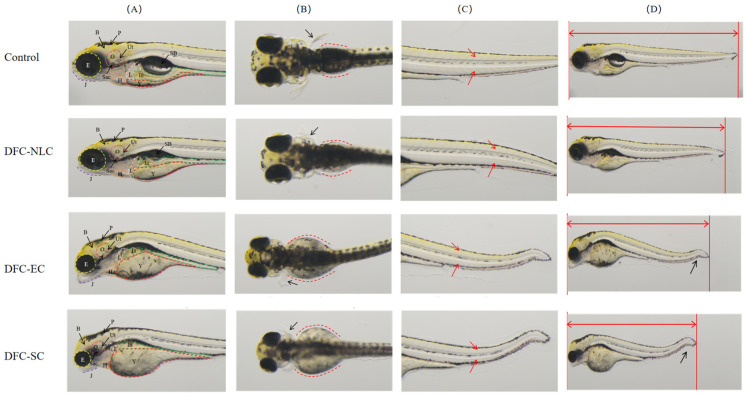
Developmental toxicity of DFC exposure to zebrafish larvae. (**A**) The representative images of zebrafish developmental toxicity. H, heart; B, brain; P, pigmentation; O, otic vesicles (Ut, utricle; and Sac, saccule); J, jaw; In, intestine; L, liver; Y, yolk sac; E, eyes; and SB, swim bladder. Representative images of fins, kidneys (**B**), muscle (**C**), and body length (**D**) in zebrafish.

Notably, with the exception of reduced eye size, yolk sac absorption delay, intestinal deformity, and absence of swim bladder, the DFC-NLC group exhibited significantly lower incidence rates of these abnormalities compared to the DFC-EC and DFC-SC groups (*p* < 0.05). These findings indicated that DFC-NLC demonstrated superior safety profiles compared to DFC-EC and DFC-SC. Typical images are shown in Figure 6.

The applications of nanopesticides are closely related to the environment and human food chains, so environmental ecological risks need to be evaluated. It has been proposed that the impacts of nanocarriers on environment should be studied [52]. By summarizing the above experimental results, the DFC-NLC was relatively safer than the commercial pesticide formulations. Some studies confirmed that negatively charged nanopesticides had low cytotoxicity to cells [53,54,55]. In addition, a water-based DFC-NLC pesticide system, which only contains water, solid–liquid lipid, and active ingredient DFC, is more environmentally friendly. The commercial DFC products with similar properties usually include organic solvents and various surfactants at relatively high concentrations. The lower toxicity of the DFC-NLC may attribute to the absence of organic solvents and to its being a relatively safe water-based system [56].

## 3. Materials and Methods

### 3.1. Materials

#### 3.1.1. Chemicals and Reagents

Monostearin, stearic acid, tristearin, and behenic acid glyceride were provided by Shanghai Macklin Biochemical Co., Ltd. (Shanghai, China). Oleic acid and poloxamer 188 were acquired from NOF Corporation (Ebisu, Shibuya-ku, Tokyo, Japan) and Jiqi Pharmaceutical Co., Ltd., Shenyang Pharmaceutical University (Jilin, China), respectively. Methanol (guarantee reagent grade) and formic acid were sourced from Merck (Darmstadt, Germany) and Tedia company (Fairfield, CT, USA). FITC and ODA were purchased from Acros Organic (New York, NJ, USA) and Fluka Analytical (Waltham, MA, USA). DFC and its commercial formulations (25% DFC-EC, 40% DFC-SC) were obtained from Shanghai Pesticide Research Institute Co., Ltd. (Shanghai, China), Syngenta Crop Protection Co., Ltd. (Basel, Switzerland), and Limin Chemical Co., Ltd. (Xinyi, China). Stock solutions of DFC (1000 mg·L^−1^) were prepared in ethanol and diluted to working concentrations (stored at 4 °C). All other reagents were of analytical grade from commercial sources.

#### 3.1.2. Biological Materials

Rhizoctonia solani was procured from the Agricultural Culture Collection of China and preserved on PDA slants (4 °C). Wild-type AB strain zebrafish embryos (6 h post-fertilization) were supplied by Hangzhou Hunter Biotechnology Co., Ltd. (Hangzhou, China). Zebrafish were maintained in automated systems under controlled conditions: 28 °C, pH 6.5~8.5, salinity 0.02%, conductivity 450~550 μs·cm^−1^.

### 3.2. Preparation of DFC-NLC Dispersions

The preparation protocol for drug-encapsulated NLCs was established in our prior research [57]. The preparation was carried out in a sterile environment in order to minimize risks of microbial, particulate, and endotoxin/pyrogen contamination. Lipid formations were as follows: (1) monostearin, stearic acid, tristearin, and behenic acid glyceride (18 mg) were used as solid lipid materials, and oleic acid (2 mg) was used as liquid lipid material; and (2) monostearin (18 mg, 16 mg, 14 mg) and oleic acid (2 mg, 4 mg, 6 mg) were mixed to obtain 20 mg solid–liquid lipid, yielding oleic acid proportions of 10%, 20%, and 30%, respectively.

DFC-NLC dispersions were prepared by solvent diffusion method. The 2 mg DFC and above lipids were dissolved in 2 mL of warm ethanol, respectively. This organic phase was rapidly injected into preheated distilled water (20 mL, 70 °C) under mechanical stirring at 400 rpm for 5 min (DC-40, Hangzhou Electrical Engineering Instruments, Hangzhou, China). Then, it was cooled to room temperature and acidified to pH 1.20 using 0.1 M HCl to induce aggregation. The precipitated NLC was collected by the first centrifugation at 10,000× *g* for 15 min (5430R, eppendorf, Hamburg, Germany), and the unentrapped drug was separated in the supernate. The NLC precipitate was resuspended in 20 mL distilled water containing 0.1% (*w*/*v*) poloxamer 188 by probe ultrasonication (JY92-II, Scientz Biotechnology Co., Ltd., Ningbo, China) using 20 burst cycles (200 W, active every 2 s for a 3 s duration). Then, the resultant dispersion was collected for the following studies.

Blank NLC dispersion was prepared identically without drug incorporation.

### 3.3. Particle Size and Zeta Potential Measurements

NLC dispersion (blank or drug-loaded) was diluted 20 times with distilled water prior to analysis. Average diameter, polydispersity index, and zeta potential were assessed using a Zetasizer analyzer (3000HS, Malvern Instruments, Worcestershire, UK).

Stability test for short-term laboratory applications of DFC-NLC dispersion was evaluated after 5-day storage under 4 °C in the dark, which was supported by the comparison of particle size, polydispersity index, and zeta potential.

### 3.4. TEM Observation

The morphological observation of DFC-NLC was performed by TEM (TECNAI 10, PHILIPS, Amsterdam, The Netherlands). Samples for TEM analysis were prepared by placing about 20 μL of DFC-NLC dispersions on the carbon-coated copper grid and then drying at room temperature before transfer to the TEM sample chamber.

### 3.5. Drug Entrapment Efficiency and Drug Loading Determination

#### 3.5.1. HPLC-MS/MS Analysis

NLC dispersion was treated with the second centrifugation (10,000× *g*, 15 min). The supernates obtained from two centrifugations were quantified using HPLC-MS/MS, as detailed below.

HPLC-MS/MS analysis was performed on Agilent HPLC system coupled to Agilent 6490 triple quadrupole mass spectrometer equipped with electrospray ionization (ESI) source (Agilent, Palo Alto, CA, USA). Chromatographic separation was performed by injecting 1 μL on an analytical RRHD Eclipse Plus C_18_ column (1.8 μm, 2.1 mm × 50 mm, Agilent, USA). The column temperature was kept at 30 °C. The mobile phase consisted of 0.1% formic acid in methanol (solvent A, 15% by volume) and 0.1% formic acid solution (solvent B, 85% by volume). The flow rate was set at 0.2 mL·min^−1^. The total running time was 5 min. Electrospray in positive mode was used, and the spray voltage was 3.0 kV. The capillary temperature was 350 °C. Auxiliary gas and sheath gas were normal nitrogen. Collision gas was high pure argon with pressure at 0.2 Pa in collision cell. Table 6 represents the MS/MS transitions selected for quantification and confirmation together with the optimized parameters for DFC. The retention time of DFC was about 1.5 min.

#### 3.5.2. Calculations

The drug entrapment efficiency and drug loading of DFC-NLC were calculated from Equations (1) and (2):

Drug entrapment efficiency (%) = (Wa − W_sl_ − W_s2_)/Wa × 100% (1)

Drug loading (%) = (Wa − W_s1_ − W_s2_)/(Wa − W_s1_ − W_s2_ + W_L_) × 100% (2)

Wa: Total drug quantity added to the system; W_s1_: unencapsulated drug quantified in the first supernate; W_s2_: unencapsulated drug in the second supernate; and W_L_: total lipid mass introduced into the formulation.

### 3.6. Antifungal Activity of DFC-NLC Dispersion

The antifungal activity of DFC-NLC dispersion against rice sheath blight was assessed through mycelial growth inhibition assays. *R. solani* as model, PDA was employed to grow mycelia discs after 48 h of culturing at 25 °C. The discs of mycelial fungi were taken from the colony margins. DFC-NLC dispersions and commercial dosage forms, such as 25% DFC-EC and 40% DFC-SC, were added to PDA medium containing 1 mM salicylhydroxamic acid to make a series of effective DFC concentrations (1.0, 0.5, 0.25, 0.125, 0.0625, 0.0313, and 0.0156 mg·L^−1^). After incubating the treated Petri dishes at 25 °C, the colony growth diameter was measured after 48 h of culturing using a cross method. Data were analyzed using a primary model by plotting colony diameter against the commercial dosage forms (DFC-EC and DFC-SC), blank NLC dispersions, and distilled water. The percentage of mycelial growth inhibition was quantified, and EC_50_ was determined through dose-response regression analysis.

### 3.7. Targeting Dynamic Distributions

#### 3.7.1. Synthesis of ODA-FITC

The synthetic method for ODA-FITC was detailed in prior research [58]. Briefly, 20 mg ODA was added in 5 mL ethanol and then treated with ultrasonically assisted dissolution (Sonic Purger CQ250, Academy of Shanghai Shipping Electric Instrument). A total of 28 mg FITC was added under dark and magnetic stirring (400 rpm) into mentioned ethanol solution and reacted at room temperature for 24 h to obtain ODA-FITC. Appropriate amount of water was added to precipitate ODA-FITC graft. The precipitate was collected by filtration with 0.45 μm Millipore filter and washed twice by 20 mL distilled water. The final product was lyophilized and stored in dark for further use.

#### 3.7.2. Preparation of FITC-Labeled DFC-NLC Dispersion

As solid lipid, 4.5 mg ODA-FITC graft was added into the lipid organic phase and then dispersed into 20 mL distilled water via mechanical agitation at 400 rpm for 5 min under thermostatic conditions at 70 °C in a water bath. FITC-labeled DFC-NLC dispersion was prepared by the same method as mentioned in “Section 3.2”.

#### 3.7.3. Pathogen Uptakes of FITC-Labeled DFC-NLCs

The cellular uptake of FITC-labeled DFC-NLCs was assessed as follows. Firstly, microscopy glass slide was sterilized by dry heat sterilization at 160 °C for 90 min after cleaning with sterilized water twice. *R. solani* was cultured on PDA plate at 25 °C for 2 days during vigorous growth. The mycelial discs (2 mm in diameter) of actively growing fungal pathogen were taken from the colony margins and aseptically placed in the center of nutrient PDA culture medium. Then, 1 mL of FITC-labeled DFC-NLC dispersion was added. The sterile microscopy glass slide was quickly inserted into the culture medium and incubated at 25 °C for 48 h. When the mycelium grew to 1/2 of the slide, the slide was taken out, and a drop of sealing solution (0.5 mol·L^−1^ phosphate buffer mixed with glycerol) was added. Pathogen uptakes of FITC-labeled DFC-NLCs were evaluated under an inverted fluorescence microscope (Axio Vert A1, ZEISS, Jena, Germany).

### 3.8. Safety Assessment

#### 3.8.1. Acute Toxicity Assessment

Thirty wild-type AB strain zebrafish were distributed into 6-well plates (Bioland Biotechnology Co., Ltd., Huzhou, China) in 3 mL fresh fish water. Zebrafish were exposed to aqueous solutions of DFC-NLC, DFC-EC, and DFC-SC with series concentrations (DFC-NLC at DFC concentrations of 5.00, 6.25, 7.50, 8.75, and 10.0 mg·L^−1^; DFC-EC and DFC-SC at DFC concentrations of 1.25, 2.50, 5.00, 10.0, and 20.0 mg·L^−1^), while a normal control group was maintained under identical conditions. All groups were incubated at 28 °C for 5 dpt under standardized husbandry conditions. Mortality events were recorded, and dead zebrafish were immediately removed. Mortality curves were generated using Origin 8.0 (OriginLab, San Francisco, CA, USA). MNLC and LC_10_ were calculated with logistic regression [59].

#### 3.8.2. Developmental Toxicity Assessment

The zebrafish were exposed to aqueous solutions of DFC-NLC, DFC-EC, and DFC-SC (each at a concentration of 4.53 mg·L^−1^ DFC). The embryos were observed under the dissecting microscope (SZX7, OLYMPUS, Tokyo, Japan) at 5 dpt and photographed with the digital camera. The following parameters were evaluated: heart, circulatory system (hemorrhage/thrombosis), brain, jaw, eyes, liver, yolk sac, kidneys, intestine, trunk/tail/notochord, muscle/somites, fins, body length, behaviors, body pigmentation, otic vesicles, and swim bladder [60]. Representative images of affected organs were captured. Toxicity was evaluated based on the incidence of abnormalities in each organ system.

### 3.9. Statistical Analyses

All statistical analyses were performed using SPSS 13.0. Data were expressed as mean ± standard deviation (SD). For antifungal activity assays and acute toxicity evaluations, differences among groups were analyzed by one-way analysis of variance (ANOVA) followed by Duncan’s multiple comparison test. Developmental toxicity data were analyzed using the Chi-square test (χ^2^), and *p* < 0.05 was considered statistically significant.

## 4. Conclusions

This study focused on the preparation, characterization, and evaluation of antifungal efficacy and safety of DFC-NLC. DFC-NLC with different solid–liquid lipid ratios were prepared by solvent diffusion method. DFC-NLC dispersion was in nanometer range (about 200 nm), with high zeta potential and spherical shape, with encapsulation efficiency of 69.1 ± 1.8%~95.0 ± 2.6% and drug loading of 7.1 ± 0.3%~9.7 ± 0.6%. The DFC-NLC dispersions exhibited higher fungicidal efficacy against *R. solani* compared with DFC-EC and DFC-SC (*p* < 0.05). FITC-labeled NLCs could be ingested by pathogens. Acute toxicity experiments on zebrafish indicated that the DFC-NLC was relatively safer than the commercial DFC products (*p* < 0.05). That is to say, NLC is an ideal carrier to significantly enhance the biological activity of pesticides with safer environmental risks.

## Figures and Tables

**Table 1:** Comparison of DFC-NLC dispersions formed by different solid–liquid lipid materials (mean ± SD, *n* = 3).

Solid Lipid	Liquid Lipid	Average Diameter (nm)	Polydispersity Index	Zeta Potential (mv)	Short-Term Laboratory Storage Stability
Monostearin	Oleic acid	201.0 ± 2.0	0.21 ± 0.05	−22.1 ± 0.5	Keep stable
Stearic acid	Oleic acid	212.3 ± 2.7	0.35 ± 0.08	−15.4 ± 0.4	Tend to coagulate or flocculate
Tristearin	Oleic acid	176.1 ± 3.4	0.29 ± 0.06	−18.6 ± 0.3	
Behenic acid glyceride	Oleic acid	180.6 ± 3.2	0.30 ± 0.05	−17.9 ± 0.3	

**Table 2:** Characteristics of DFC-NLC dispersions formed by different solid–liquid lipid ratios with oleic acid contents 10%, 20%, and 30% (mean ± SD, *n* = 3).

Monostearin Proportion (%)	Oleic Acid Proportion (%)	Average Diameter (nm)	Polydispersity Index	Zeta Potential (mv)	Entrapment Efficiency (%)	Drug Loading (%)
90	10	201.0 ± 3.2	0.20 ± 0.05	−22.1 ± 0.2	69.1 ± 1.8	7.1 ± 0.3
80	20	198.3 ± 2.6	0.20 ± 0.04	−23.4 ± 0.4	78.9 ± 2.3	8.1 ± 0.5
70	30	176.1 ± 2.8	0.18 ± 0.06	−25.3 ± 0.3	95.0 ± 2.6	9.7 ± 0.6

**Table 3:** Fungicidal activity of DFC-NLC dispersions, DFC-EC, and DFC-SC against *R. solani*.

Samples	Regression Equation	R^2^	EC_50_ (mg·L^−1^)	95% Confidence Limits (mg·L^−1^*)*
DFC-NLC	Y = 6.0510 + 1.0823 X	0.9367	0.107 a	0.0677~0.1688
DFC-EC	Y = 5.7140 + 1.0574 X	0.9878	0.211 b	0.1721~0.2593
DFC-SC	Y = 5.5351 + 1.0838 X	0.9791	0.321 b	0.2364~0.4353

Note: Different letters in the same column indicate the statistically significant differences at the level of significance α = 0.05.

**Table 4:** Acute toxicity of DFC on zebrafish (*n* = 30).

Group	Concentration (mg·L^−1^)	Number of Deaths (Individuals)	Mortality Percentage (%)
Control	-	0	0
DFC-NLC	5.00	0	0
	6.25	5	17
	7.50	30	100
	8.75	30	100
	10.0	30	100
DFC-EC	1.25	0	0
	2.50	0	0
	5.00	10	33
	10.0	30	100
	20.0	30	100
DFC-SC	1.25	0	0
	2.50	0	0
	5.00	18	60
	10.0	30	100
	20.0	30	100

**Table 5:** Developmental toxicity of DFC on zebrafish during 5 days post treatment (dpt) (*n* = 30 per group).

Type		Abnormality Percentages (%)			
		Control	DFC-NLC	DFC-EC	DFC-SC
Heart	Pericardial edema	-	-	-	-
	Arrhythmia	-	33a	93b	100b
Circulatory system	Accelerated blood flow	-	-	-	-
	Slowed blood flow/Absence	-	33a	93b	100b
Hemorrhage/Thrombosis		-	-	-	-
Brain	Degeneration	-	-	-	-
	Reduction	-	-	-	-
Jaw	Malformation	-	47a	93b	100b
Eyes	Reduction	-	90	100	100
Liver	Enlargement	-	-	-	-
	Reduction/Absence	-	30a	63b	77b
	Degeneration	-	-	-	-
Yolk sac absorption delay		-	100	100	100
Kidneys	Pronephric edema	-	-	-	-
Intestine	Deformity	-	-a	10a	57b
Trunk/Tail/ Notochord	Curvature	-	-a	47b	90c
	Edema	-	-	-	-
Fins	Absence	-	-	-	-
Body length	Decrease	-	80a	100b	100b
Behaviors	Abnormality	-	10a	60b	100c
Muscle	Muscle degeneration	-	-	-	-
Body pigmentation		-	-	-	-
Otic vesicles	Reduction	-	63a	100b	100b
Swim bladder	Loss	-	97	100	100

Note: ‘-’ indicates no significant abnormalities observed; values represent abnormality percentages (%); and different letters in the same row indicate the statistically significant differences at the level of significance α = 0.05.

**Table 6:** HPLC-MS/MS conditions for DFC.

Compound	Parent Mass (*m*/*z*)	Product Mass (*m*/*z*)	Collision Energy (V)
DFC	406	337	29
		251 *	25

Note: * Quantitative ion.

## Data Availability

The data presented in this study are available in this article.
